# Supplementary figures and images for: Temporal Dynamics of Pulmonary Fibrosis and Immune Dysregulation in a Collagen V-Driven Systemic Sclerosis Model
Source: Int J Mol Sci. 2025 Dec 24;27(1):197. doi: 10.3390/ijms27010197 (PMC12786071; doi:10.3390/ijms27010197)

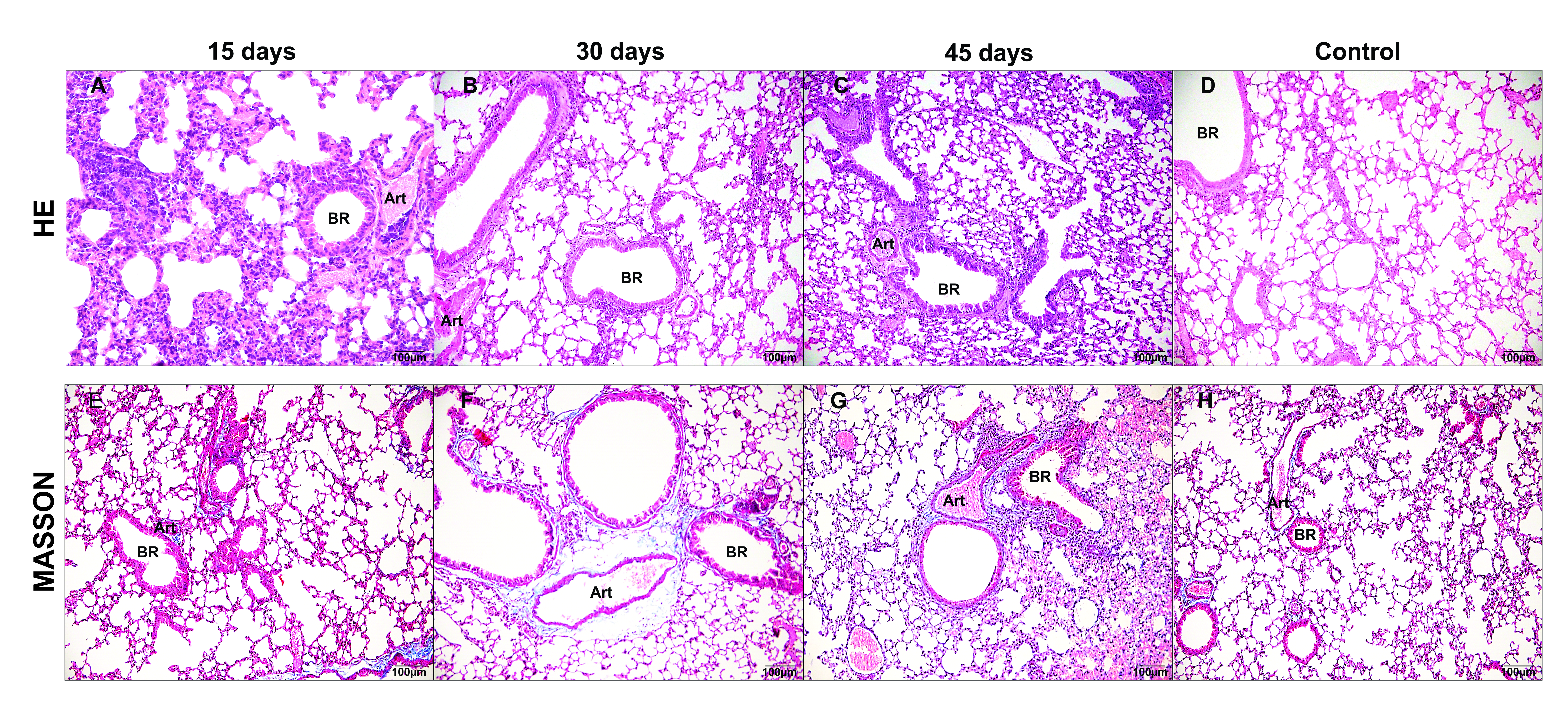

Supplement: Supplementary file 1 [file ijms-27-00197-s001.zip › Supplementary Figure S1.jpg]

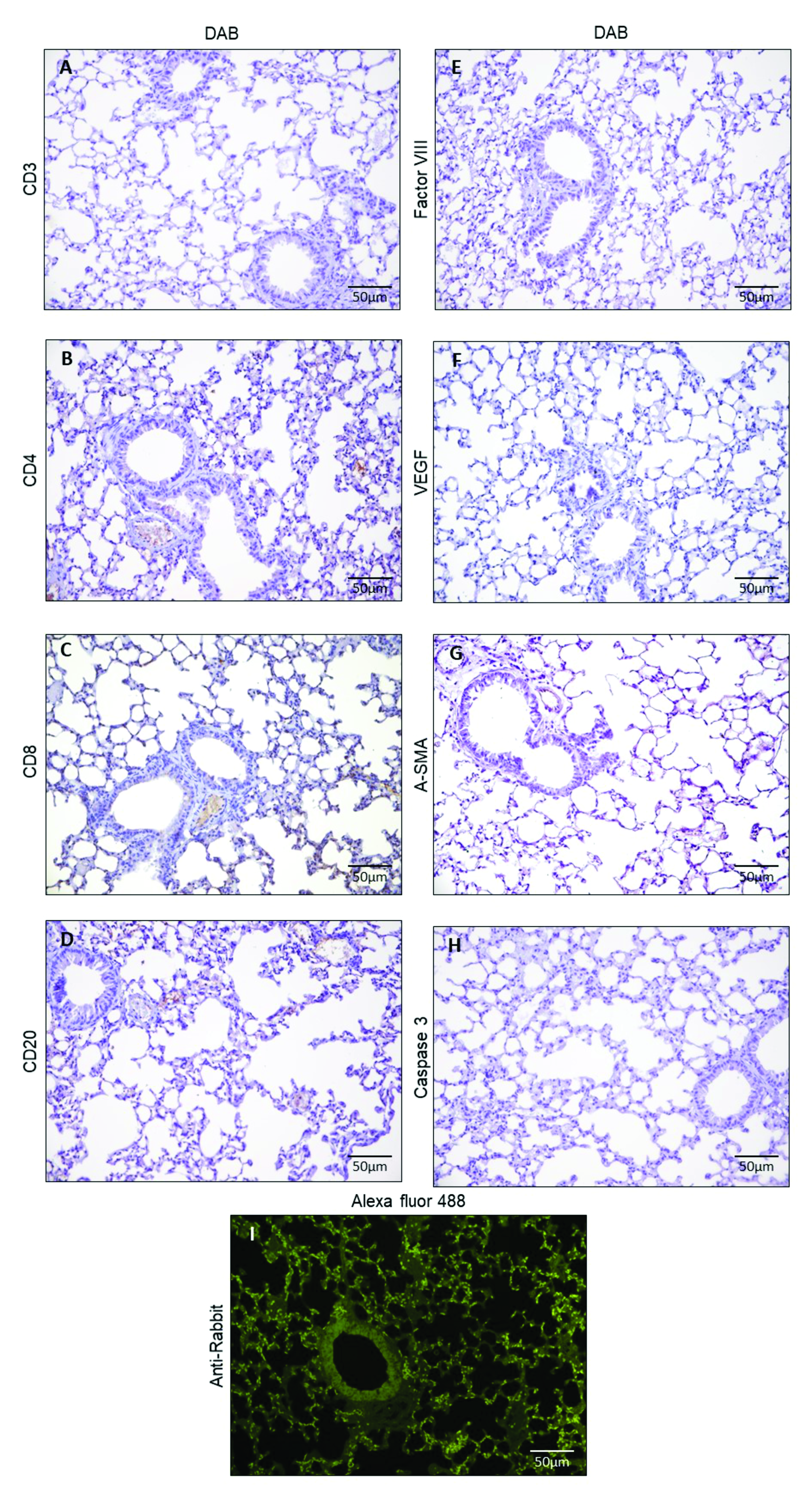

Supplement: Supplementary file 1 [file ijms-27-00197-s001.zip › Supplementary Figure S2.jpg]
